# Artificial intelligence in glaucoma: opportunities, challenges, and future directions

**DOI:** 10.1186/s12938-023-01187-8

**Published:** 2023-12-16

**Authors:** Xiaoqin Huang, Md Rafiqul Islam, Shanjita Akter, Fuad Ahmed, Ehsan Kazami, Hashem Abu Serhan, Alaa Abd-alrazaq, Siamak Yousefi

**Affiliations:** 1 Department of Ophthalmology, University of Tennessee Health Science Center, Memphis, USA; 2 Business Information Systems, Australian Institute of Higher Education, Sydney, Australia; 3 School of Computer Science, Taylors University, Subang Jaya, Malaysia; 4 Department of Computer Science & Engineering, Islamic University of Technology (IUT), Gazipur, Bangladesh; 5 Ophthalmology, General Hospital of Mahabad, Urmia University of Medical Sciences, Urmia, Iran; 6 Department of Ophthalmology, Hamad Medical Corporations, Doha, Qatar; 7 AI Center for Precision Health, Weill Cornell Medicine-Qatar, Doha, Qatar; 8 Department of Genetics, Genomics, and Informatics, University of Tennessee Health Science Center, Memphis, USA

**Keywords:** Artificial intelligence, Glaucoma, Machine learning, Deep learning

## Abstract

Artificial intelligence (AI) has shown excellent diagnostic performance in detecting various complex problems related to many areas of healthcare including ophthalmology. AI diagnostic systems developed from fundus images have become state-of-the-art tools in diagnosing retinal conditions and glaucoma as well as other ocular diseases. However, designing and implementing AI models using large imaging data is challenging. In this study, we review different machine learning (ML) and deep learning (DL) techniques applied to multiple modalities of retinal data, such as fundus images and visual fields for glaucoma detection, progression assessment, staging and so on. We summarize findings and provide several taxonomies to help the reader understand the evolution of conventional and emerging AI models in glaucoma. We discuss opportunities and challenges facing AI application in glaucoma and highlight some key themes from the existing literature that may help to explore future studies. Our goal in this systematic review is to help readers and researchers to understand critical aspects of AI related to glaucoma as well as determine the necessary steps and requirements for the successful development of AI models in glaucoma.

## Introduction

Glaucoma is an optic neuropathy accompanied by characteristic structural and functional changes [1]. It affects over 90 million individuals worldwide and constitutes the second leading cause of blindness and subsequent disability overall [2, 3]. The number of people aged 40–80 years with glaucoma worldwide was estimated to be 64.3 million in 2013, with projections that this number will increase to 76.0 million in 2020, and 111.8 million in 2040 [4]. Because older people make up the fastest-growing part of the US population, glaucoma will become even more prevalent in the US in the coming decades. As such, population-based screening for glaucoma becomes critical.

Glaucoma detection is challenging particularly at the early stages of the disease; however, early detection may lead to slowing its progression and future vision loss [5]. A major challenge in detecting glaucoma is that signs and symptoms may manifest only when significant vision has been already lost. Therefore, it is critical to diagnose glaucoma early to prevent future vision loss [6]. Glaucoma diagnosis typically includes assessment of the optic nerve head (ONH) through retinal examination, intraocular pressure (IOP) measurement, evaluation of visual fields (VFs), and examining other related factors.

Assessing the ONH in glaucoma is an important diagnostic step, as the primary implication for glaucoma is glaucomatous optic neuropathy (GON), which is widely identified through fundus photographs or optical coherence tomography (OCT) images. Currently, fundus photography is better suited for glaucoma screening because fundus cameras are affordable and more importantly, portable. However, development of low-cost and portable OCT systems is advancing as well, and these portable OCT technologies are poised to gain popularity in the coming years. Fundus photography has been the most established modality for documenting the status of the optic nerve and detecting GON since 1857. As a result, large, annotated datasets of fundus photographs are currently available and importantly, they are appropriate for machine learning (ML) and deep learning (DL) models. In contrast, clinical evaluations of the ONH are subjective and prone to error. According to prior research, it has been reported that both ophthalmology trainees and comprehensive ophthalmologists underestimated the likelihood of glaucoma in 20% of disc photographs. Additionally, ophthalmology trainees and comprehensive ophthalmologists were twice as likely to underestimate or overestimate the likelihood of glaucoma due to various factors, such as the variability in cup-to-disc ratio, peripapillary RNFL atrophy, and the presence of disc hemorrhage [7]. Optic nerve assessment is primarily performed in a subjective manner while most of the useful structural and functional features of healthy and diseased patients are overlapping therefore leading to inter- and intra-observer variability. The problem is worse for monitoring as glaucoma progression is often slow and happens over decades, making prediction of progression highly challenging. As such, automated ML models may provide more objective, consistent, and more accurate outcomes.

Artificial intelligence (AI) and DL models are emerging areas that automate the interpretation of retinal images. Advancements in computer systems and availability of large datasets and algorithms allow these systems to mimic human thought processes such as learning, reasoning, and self-correction. DL, a subfield of ML and AI, has made significant progress over the past few years and development of objective systems to automate glaucoma detection has been highly promising [8]. Although many studies demonstrated promising results in detecting glaucoma using AI, limitations still exist in many perspectives. For example, lack of standardized and consensus glaucoma definitions makes it difficult to evaluate the results consistently in different datasets; the shortage of large, well-annotated datasets of good quality limited the generalizability of AI model; the limited interpretability and liability of the DL model hurdled the implementation of it in clinic.

This review aims to identify ML-based models applied to glaucoma over the past few years and create a reference of those models and their performance. We also generate several taxonomies including categories of ML models, input data types, and level of performance and compare different ML types to identify better performing approaches. Lastly, we provide insight into current challenges and future directions as well.

The paper is organized as follows:

This paragraph ends the introductory section. An overview of AI in glaucoma is presented in Section “Literature review”. Section "Overview of the AI models in glaucoma" outlines applications of specific AI models in glaucoma based on various categories. Section "Discussion” presents open issues and future directions of AI in glaucoma. Finally, Section “Conclusions” concludes this review. Section "Methods” presents literature search and filtering strategies for this review.

## Literature review

### Glaucoma

Glaucoma is a group of heterogenous diseases that may lead to irreversible vision loss [1]. In some forms of glaucoma, increased IOP impacts the retina and ONH, which in turn, may lead to irreversible vision loss [9]. Lowering IOP is a proven treatment for open-angle glaucoma (OAG).

Although glaucoma detection is challenging, particularly at the early stages of the disease, early detection is critical in order to provide timely treatments that may be effective in slowing its progression [5]. Glaucoma is typically diagnosed by evaluating the ONH and the adjacent retinal nerve fiber layer (RNFL) through retinal examination and imaging tools, assessing VFs, and evaluating IOP levels.

### AI for glaucoma

**Fig. 1:**
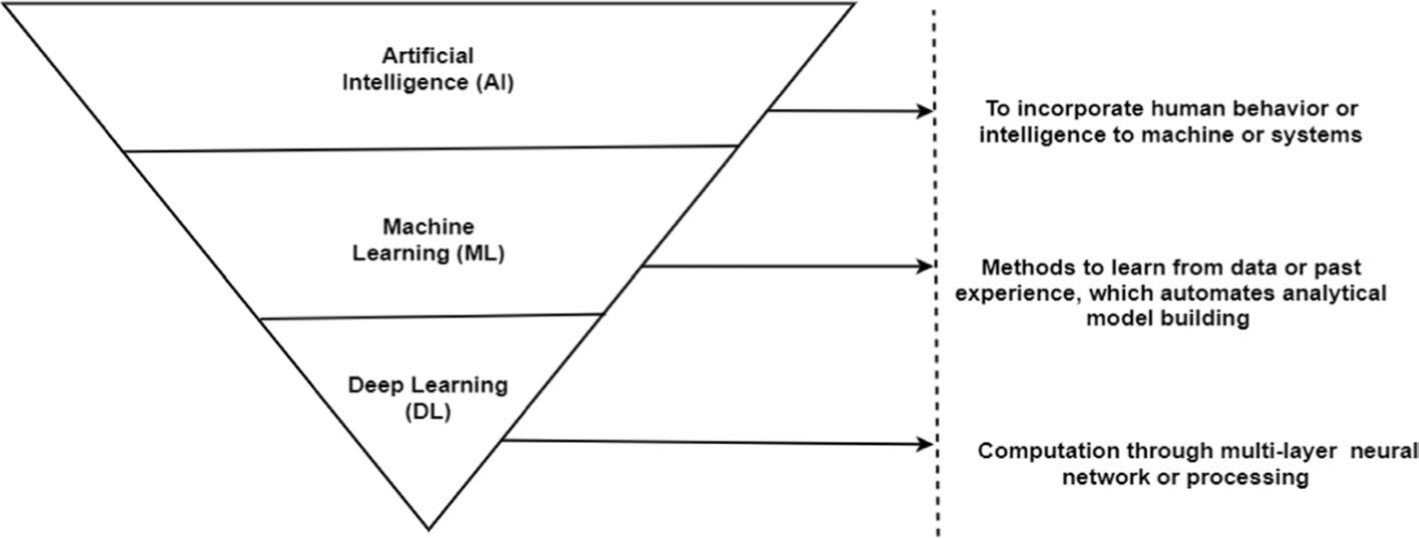
Illustration and definition of artificial intelligence (AI), machine learning (ML), and deep learning (DL)

Not only is glaucoma diagnosis potentially time-consuming and costly, but also its reliance on an individual clinician’s knowledge and ability makes it subjective and prone to over/under estimation [10]. Alternatively, automated AI models could minimize subjectivity by interpreting and quantifying retinal and optic nerve images. AI has several other applications for glaucoma. For instance, AI can be used to optimize workflows and processes in glaucoma clinics that may lead to more time for clinicians to interact with patients thus enhancing overall care. AI could be used to quantify optic cup, disc, and rim characteristics in fundus images, retinal layers in OCT images, and patterns of VF loss. Such applications hold promise for providing improved glaucoma assessment, as well as forecasting, screening, diagnosing, and prognosing glaucoma. Figure 1 describes the overall AI domain and its subcategories including ML and DL. While DL models are more appropriate for analysis of image-based glaucoma data, other categories of ML models may be more appropriate for VF and other non-image data. Different categories of AI models including expert systems may be utilized to optimize glaucoma clinic workflows and processes.

## Overview of the AI models in glaucoma

**Fig. 2:**
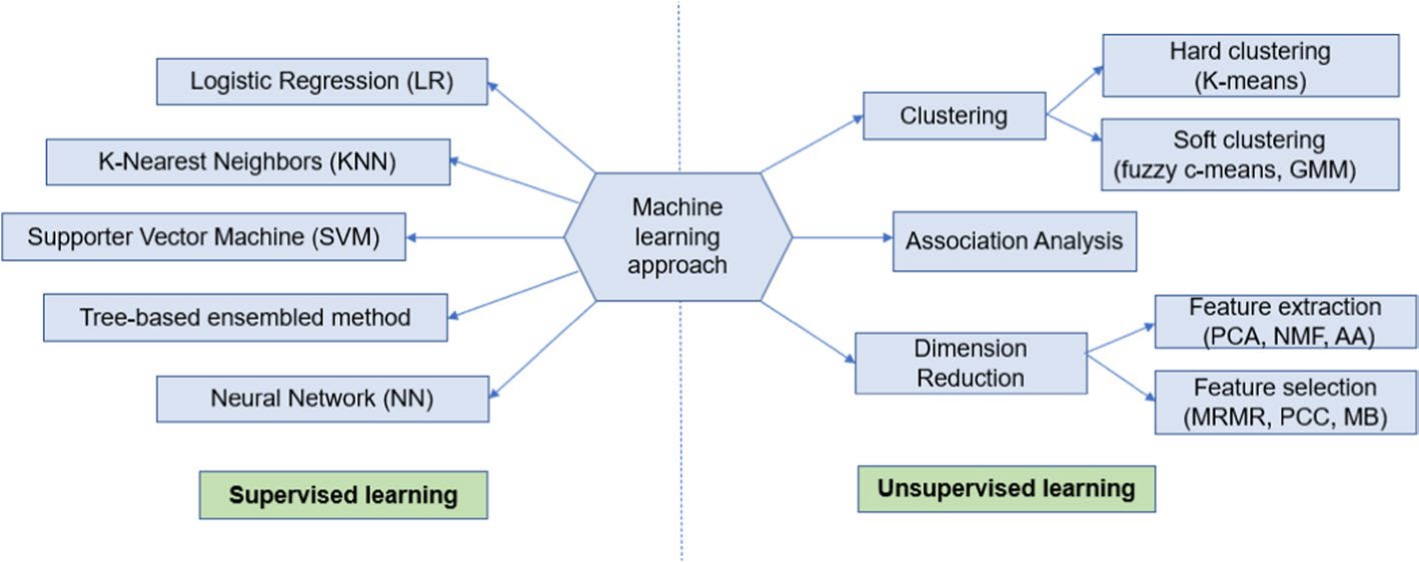
Various types of ML models applied to glaucoma. GMM: Gaussian Mixture Modeling; PCA: Principal Component Analysis; NMF: Non-negative Matrix Factorization; AA: Archetypal Analysis; PCC: Pearson Correlation Coefficient, MB: Markov Blanket; mRMR: Minimum Redundancy Maximum Relevance

This section reviews different AI models from the literature that have been used for glaucoma assessment. Figure 2 shows different ML-based models that have been applied to detect glaucoma. These models have been grouped into two major categories including supervised and unsupervised learning models. In supervised learning, the model is trained on labeled data with each data instance having a known outcome. Unsupervised learning describes algorithms for finding patterns in data, without prior knowledge of outcomes for data instances [11]. Based on this taxonomy, we will highlight some of the representative ML studies applied to glaucoma and provide their strengths and limitations.

### Machine learning

#### Supervised machine learning

Supervised ML [12–80] has been widely used in glaucoma detection, severity classification, progression prediction, segmentation, etc., based on different modalities, such as VF, fundus, OCT, clinical data, transcriptomic data, etc.

Several metrics were used for model evaluation, such as: accuracy (the proportion of correctly classified samples relative to the total number of samples); sensitivity/recall (the rate of positive samples correctly classified, reflecting the ratio of correctly classified positive samples to all samples assigned to the positive class); specificity (measures the rate of negative samples correctly classified, determined by the ratio of correctly classified negative samples to all samples belonging to the negative class); error rates (the ratio of the incorrectly classified samples to the total number of samples); precision (the proportion of true positive predictions out of all positive predictions from the model); true positive rate (TPR, the proportion of actual positive samples that the model correctly identified as positive); false positive rate (FPR, the proportion of actual negative samples that the model incorrectly identified as positive); area under the receiver operating characteristic (ROC) curve (AUC, the model’s performance across various thresholds, presenting TPR against FPR at various threshold settings), area under precision—recall curve (AUPRC, the classification model performance appropriate for imbalanced classes, demonstrating the precision against the recall at different threshold settings).

Logistic regression (LR) is a supervised model designed to estimate the probability between categorical classes. LR has also been used in glaucoma diagnosis in various studies. Lu et al. [28] used four ML classifiers to detect glaucoma based on biomechanical data from 52 patients including 20 glaucoma (40 eyes) and 32 healthy (64 eyes). The LR model obtained the best accuracy of 0.983, AUC of 0.990, sensitivity of 98.9% (at 80% specificity), and sensitivity of 97.7% (at 95% specificity) based on 3-fold cross-validation (CV). Baxter et al. [35] developed machine models to predict the requirement for surgical intervention in individuals diagnosed with primary open-angle glaucoma (POAG) utilizing systemic parameters obtained from the electronic health records (EHR) system from 385 POAG patients. They used leave-one-out CV, and the best model was multivariable LR with an AUC of 0.67. This model also provided the odds ratio of the factors which were associated with the risk of glaucoma surgery. Higher mean systolic blood pressure (OR: 1.09, *P* < 0.001) and use of anticoagulant medication (OR: 2.75, p = 0.042) were significantly associated with increased risk of glaucoma surgery. The major advantage of the LR models is their simplicity and explainability, which would be an essential advantage in glaucoma research and clinics.

K-Nearest Neighbor (KNN) is a supervised classification technique to estimate the likelihood that a data point belonging to a specific group by analyzing the groups to which its nearest neighboring data points belong. The KNN model has been used in glaucoma detection and some of the studies obtained better results using KNN than the other models. Singh, et al. [40] developed several ML models for glaucoma diagnosis from 70 glaucomatous and 70 healthy eyes based on OCT data. They extracted 45 features from OCT images and the highest AUC of 0.97 was achieved by a KNN model tested using 5-fold CV. Singh et al. [48] also developed an interconnected architecture with Customized Particle Swarm Optimization (CPSO) and four machine-learning classifiers based on 110 fundus images and found CPSO-KNN demonstrated superior performance compared to other models, achieving an accuracy of 0.99, specificity of 0.96, sensitivity of 0.97 and precision of 0.97, F1-score of 0.97 and kappa of 0.94 by utilizing 5-fold CV.

Support Vector Machine (SVM): SVM is a supervised ML technique that has the capability to tackle both classification and regression problems. The algorithm aims to identify the optimal line or decision boundary that separates different groups, enabling accurate classification of additional data points into their respective categories. SVM classifier has been widely reported in the literature for detecting glaucoma. For instance, *Goldbaum* et al. [12] compared various ML models for glaucoma diagnosis based on Standard Automated Perimetry (SAP) data collected from 189 normal eyes and 156 glaucomatous eyes, and SVM with Gaussian kernel with the input of VF sensitivities at each of 52 locations plus age. Results from this study obtained the second highest performance with AUC of 0.903, sensitivity of 0.53 (at specificity of 1.0) and sensitivity of 0.71 (at specificity of 0.9) based on 10-fold CV. Zangwill et al. [13] employed SVM models to detect glaucoma based on Heidelberg Retina Tomograph (HRT) data collected from 95 glaucomatous eyes and 135 normal eyes, and obtained the best performance with AUC of 0.964, sensitivity of 97% (at 75% specificity), and sensitivity of 85% (at 90% specificity) with the input of all parameters combined, including RNFL regional and global parameters, sectoral mean height contour along the disc margin, sectoral parapapillary mean height contour, sectoral RNFL thickness along the disc margin, and sectoral parapapillary RNFL thickness. Evaluations were performed based on 10-fold CV. Burgansky-Eliash et al. [14] developed different ML models for glaucoma detection from 47 glaucomatous eyes and 42 healthy eyes based on OCT parameters and found SVM with eight OCT parameters achieved the best performance with AUC of 0.981, accuracy of 0.966, sensitivity of 97.9% (at the specificity of 80%), and sensitivity of 92.5% (at specificity of 92.5%) using 6-fold CV. Townsend et al. [15] developed several ML models for glaucoma detection based on HRT3 data collected from 60 healthy subjects and 140 glaucomatous subjects. The SVM-radial applied on all HRT3 parameters showed significant improvement over the other models and obtained an AUC of 0.904, accuracy of 85.0%, sensitivity of 85.7% (at 80% specificity) and 64.8% sensitivity (at 95% specificity) based on leave-one-out CV. Garcia-Morate et al. [16] developed an SVM model to detection glaucoma using 136 glaucomatous eyes and 117 non-glaucomatous eyes based on HRT2 parameters. The SVM model exploiting 22 parameters obtained the highest performance with AUC of 0.905, sensitivity of 85.3% (at 75% specificity), and 79.4% sensitivity (at 90% specificity) using 10-fold CV. Bizios et al. [17] developed numerous AI models to detect glaucoma based on OCT-A scans collected from 90 healthy and 62 glaucomatous subjects and reported that SVM achieved the best performance with an AUC of 0.989 (95% confidence interval: 0.979–1.0) using 10-fold CV.

In summary, SVM worked well in dealing with VF, HRT, and OCT parameters to detect glaucoma mostly in earlier studies from 2002 to 2010. SVM is straightforward to implement and has relatively high explainability.

Tree-based ensembled model: The tree-based ensembled method has been reported to have better performance than a single tree-based model. Random Forest (RF), XGboost, and gradient boosting models from this family have been widely used in glaucoma diagnosis based on VFs and OCT parameters and have shown reasonable performance. Barella et al. [19] developed multiple ML models to detect early to moderate POAG from 57 early to moderate POAG and 46 healthy patients based on RNFL and optic nerve parameters collected from SD-OCT instrument. RF obtained the best AUC of 0.877 based on 13 input parameters with sensitivity of 64.9% (at 80% specificity) and sensitivity of 49.1% (at 90% specificity) using 10-fold CV. Hirasawa et al. [18] used various ML models to predict vision-related quality of life (VRQoL) based on VF and visual acuity from 164 glaucomatous patients. Based on this regression problem, RF and boosting models obtained the lowest error rate with root mean square error (RMSE) of 1.99. Silva et al. [20] developed models for glaucoma diagnosis based on SD-OCT and SAP (24–2) data collected from 62 glaucomatous patients and 48 healthy subjects. Based on four features, RF achieved the best AUC of 0.946 with the sensitivity of 95.16% (at 80% specificity), and the sensitivity of 82.25% (at 90% specificity) using 10-fold CV. Kim et al. [25] developed several ML models for diagnosis of glaucoma based on RNFL thickness and VFs collected from 399 cases and RF showed the best performance with an accuracy of 98%, sensitivity of 98.3%, specificity of 97.5%, and AUC of 0.979 when using seven features on internal testing set with 100 cases. Oh et al. [44]applied various ML models to detect glaucoma based on clinical data (IOP, OCT measurements, VF examinations) collected from 1244 eyes and observed XGboost the best performing model with an accuracy of 94.7%, sensitivity of 94.1%, specificity of 95.0%, and AUC of 0.945 with 10-fold CV.

Neural network (NN), also known as artificial neural networks (ANNs), are structured with layers of nodes and usually consist of an input layer, one or multiple hidden layers, and an output layer. Within this network, each node, also known as an artificial neuron, establishes connections with other nodes and possesses an assigned weight and threshold. When the output of a node surpasses a predetermined threshold, it becomes activated and transmits its output to the subsequent layer of the network; otherwise, no output is passed along to the next layer of the network. NNs have long been used for glaucoma-related tasks based on VFs and other imaging parameters. Omodaka et al. [24] used an NN with a structure of nine input layer units, eight hidden layer units, and four output layer units to identify the status of 163 eyes based on 15 features selected by minimum redundancy maximum relevance (mRMR) from 91 OCT parameters. The model achieved an accuracy of 87.8% (Cohen’s Kappa of 0.83) based on 10-fold CV. An et al. [27] used the same dataset and compared the performance of NN, SVM, and Naïve Bayesian models based on nine parameters selected by combining mRMR and genetic-algorithm-based feature selection. They identified the NN model as the best performing algorithm, with accuracy of 87.8% using 10-fold CV.

Overall, these conventional supervised ML models have been used widely in glaucoma diagnosis based on VFs, RNFL parameters, or other clinical factors. Among these models, we observed that SVM was used more frequently and obtained better performance, compared to other conventional ML models.

#### Unsupervised machine learning

Unsupervised learning is used for learning representative features and extracting patterns from data. Many studies applied unsupervised learning for extracting VF or RNFL loss patterns, glaucoma staging, segmentation and other features by using clustering, association analysis, dimension reduction, etc. [37, 41, 43, 49, 56, 60, 62, 81–92].

##### Clustering

We can broadly group the clustering models into either of two categories: hard or soft clustering algorithms.

Hard clustering: In hard clustering, each data point is clustered or grouped to just one cluster and not others. K-Means is a hard clustering algorithm. In K-Means, the algorithm determines the optimal initial centroid points by minimizing the sum of the squared distances between each point and its assigned centroid across all clusters. Huang et al. [87] applied K-Means clustering to identify different stages of glaucoma without any supervision by experts. They identified four severity levels based on 13,231 VFs and determined objective thresholds of − 2.2, − 8.0 and − 17.3 dB for VF mean deviation for distinguishing normal, early, moderate, and advanced stages of glaucoma. Ammal et al. [90] used K-Means to segregate optic disc (OD) and optic cup (OC) for further glaucoma diagnosis model development based on fundus images from the Retinal Fundus Images for Glaucoma Analysis (RIGA) dataset, and validated the model using another dataset with 90 images. The result of the K-Means was compared with severity levels determined by ophthalmologists on the same data set, and authors showed that the outcome was similar.

Soft clustering: In soft or fuzzy clustering, instead of putting each data points into only one cluster, the data point can be assigned to different clusters with different likelihoods. Fuzzy c-Means and Gaussian Mixture Model (GMM) are examples of soft clustering. Fuzzy-c Means: Praveena et al. [86] applied K-Means, Fuzzy c-Means (FCM) and Spatially Weighted fuzzy C-Means Clustering (SWFCM) to automatically determine the cup-to-disc ratio (CDR) from the fundus photographs of 50 normal and 50 glaucoma eyes. The K-value is determined by hill climbing algorithm. K-Means was used for segmenting OD, while FCM was used for segmenting optic cup. SWFCM was used for segmenting both OD and OC. The error rate was calculated with reference to the manually determined CDR value (considered the gold standard) provided by ophthalmologists for comparison. The mean error of the K-Means clustering method for elliptical and morphological fitting was 4.5% and 4.1%, respectively. The mean error was reduced by the FCM clustering to 3.83% and 3.52%, and the mean error was minimized to 3.06% and 1.67% using SWFCM. GMM: GMM attempts to find a mixture of multidimensional Gaussian probability distributions. Yousefi et al. [82] applied GMM to identify different patterns of VF loss. Their GMM successfully detected three distinct clusters, which included normal eyes, eyes in the early stage of glaucoma, and eyes in the advanced stage of glaucoma based on SAP VFs collected from 2085 eyes. Based on another subset with 270 eyes, they showed that GMM detected progressing eyes at an earlier stage, compared to other methods.

##### Association analysis

Association analysis: Association rule algorithms aims to find interesting relationships among variables within extensive datasets, provided they satisfy the predetermined minimum support (a threshold for determining the minimum association) and confidence level (or accuracy, minimum threshold of the correct rule or prediction) set by the user, in order to discover a pattern with strong association. Apriori algorithms, one of the association rule categories, focus on identifying frequent associations in order to unveil intriguing relationships between attributes. Al-Shamiri et al. [89] applied Apriori algorithm to discover risk factors of glaucoma based on a survey dataset from 4000 patients. Their association analysis revealed several glaucoma risk factors including family history of glaucoma, high IOP, optic nerve damage, high myopia, diabetes, hypertension, history of eye surgery, use of some medicines, psychological stress, pressure work or study-related pressure, gastroenterology disease, and chronic constipation.

##### Dimensionality reduction

A comprehensive eye examination to diagnose glaucoma typically includes collecting numerous imaging, VF, and ocular measurements, and thus provides high-dimensional datasets. A challenge is that ML models typically tend to overfit when dealing with glaucoma data and thus is not generalizable when tested on new data. However, reducing the dimensionality may address the issue by selecting a smaller subset of the features or deriving new features from a pool of features, while preserving the information of the original features as much as feasible.

Transformations: Transformation methods typically perform linear or nonlinear transformations to map a high-dimensional space of initial input data into a lower dimensional space of the features to reduce dimensionality. The widely used methods in glaucoma research are principal component analysis (PCA), archetypal analysis, and non-negative matrix factorization (NMF).

PCA: PCA [93] refers to a mathematical technique that employs an orthogonal transformation to convert a collection of observations, which may consist of correlated variables, into a new set of linearly uncorrelated variables known as principal components (PCs). Christopher et al. [29] applied PCA to structural RNFL features from RNFL thickness maps and retained 10 PCs for glaucoma diagnosis and glaucoma progression prediction based on 235 eyes and compared OCT and SAP features. The LR model based on PCA from RNFL data obtained a better performance than other available parameters such as mean cpRNFL, SAP 24–2 MD, and FDT MD in both glaucoma diagnosis (AUC of 0.95; CI 0.92–0.98) and progression prediction (AUC of 0.74; CI 0.62–0.85) using leave-one-out CV. Yousefi et al. [88] applied PCA to 52 TD values of 13,231 VFs and selected 10 PCs (explained 90% of variance in VFs) which were subsequently input to a manifold learning model, two features (dimensions) were ultimately retained. They applied unsupervised ML and identified 30 clusters based on those two features and used those clusters for glaucoma progression detection.

Archetypal Analysis: Wang et al. [83] applied Archetypal Analysis to identify the central VF patterns of loss from 13,951 VF tests (Humphery, 10–2). They discovered 17 distinct central VF patterns and noted that incorporating coefficients from central VF archetypical patterns strongly enhances the prediction of central VF loss compared to using global indices only. In another study, Wang et al. [84] employed Archetypal Analysis to identify the central VF patterns in end-stage glaucoma based on 2912 reliable VFs (10–2) and found central VF loss in end-stage glaucoma exhibits characteristic patterns that could potentially be associated with various subtypes. Nasal loss is likely the initial central VF loss, and a particular subtype of nasal loss is highly prone to progress into complete or total loss. Such explainable models may also shed light on some aspects of glaucoma pathophysiology. Follow-up studies based on deep archetypal analysis (DAA) identified more patterns of VF loss, their role in forecasting glaucoma, and their association with rapid glaucoma progression [94–96].

NMF: Wang et al. [81] utilized NMF to identify distinct patterns of the RNFL thickness (RNFLT) based on RNFLT collected from 691 eyes. NMF identified 16 distinct RNFLT patterns (RPs). Using these RPs resulted in a substantial enhancement in the prediction of VF sensitivities. The AI-based RNFLT patterns hold promise in assisting clinicians to more effectively evaluate and interpret RNFLT maps.

Feature selection: Feature selection models to identify the most optimal subset of original input variables. Various feature selection methods have been used in glaucoma research and some improved the performance of ML models. Examples include Pearson Correlation Coefficient (PCC)-based variable selection, Markov Blanket (MB) variable selection, and the minimum Redundancy Maximum Relevance (mRMR) approaches. Lee et al. [33] developed a set of ML models for glaucoma diagnosis based on VF data collected from 632 eyes and compared the result of models with different input features which were selected by PCC-based variable selection, MB variable selection, mRMR, and features extracted by PCA. By employing a combination of total deviation (TD) values, the GHT sector map, and variable selection using SVM and MB methods, the researchers achieved the highest performance, as evaluated through 5-fold CV, with an AUC of 0.912. Omodaka et al. [24] used mRMR to select 15 features from 91 quantified ocular parameters and used an NN model for OD classification based on 165 eyes of 105 POAG patients, and obtained an accuracy of 87.8%, Cohen’s kappa of 0.83 using 10 -fold CV. An et al. [27] applied a combination of mRMR and genetic algorithm-based feature selection to identify nine most valid and relevant features from a pool of 91 ocular parameters and patients’ background information of 163 glaucomatous eyes to develop ML model for classifying glaucomatous optic discs, and using the selected nine parameters yielded the highest accuracy of 87.8% with an NN model evaluated based on 10 -fold CV.

### Deep learning

Emerging DL models have been widely used in ophthalmology and many DL models have shown promising performance in glaucoma screening, diagnosis, or quantification and segmentation of fundus photographs, VFs, and OCT images [31, 34, 51, 53, 85, 97–290]. We will discuss some of the applications in glaucoma in three broad categories of discriminative, generative, and hybrid models, as shown in Fig. 3.

**Fig. 3:**
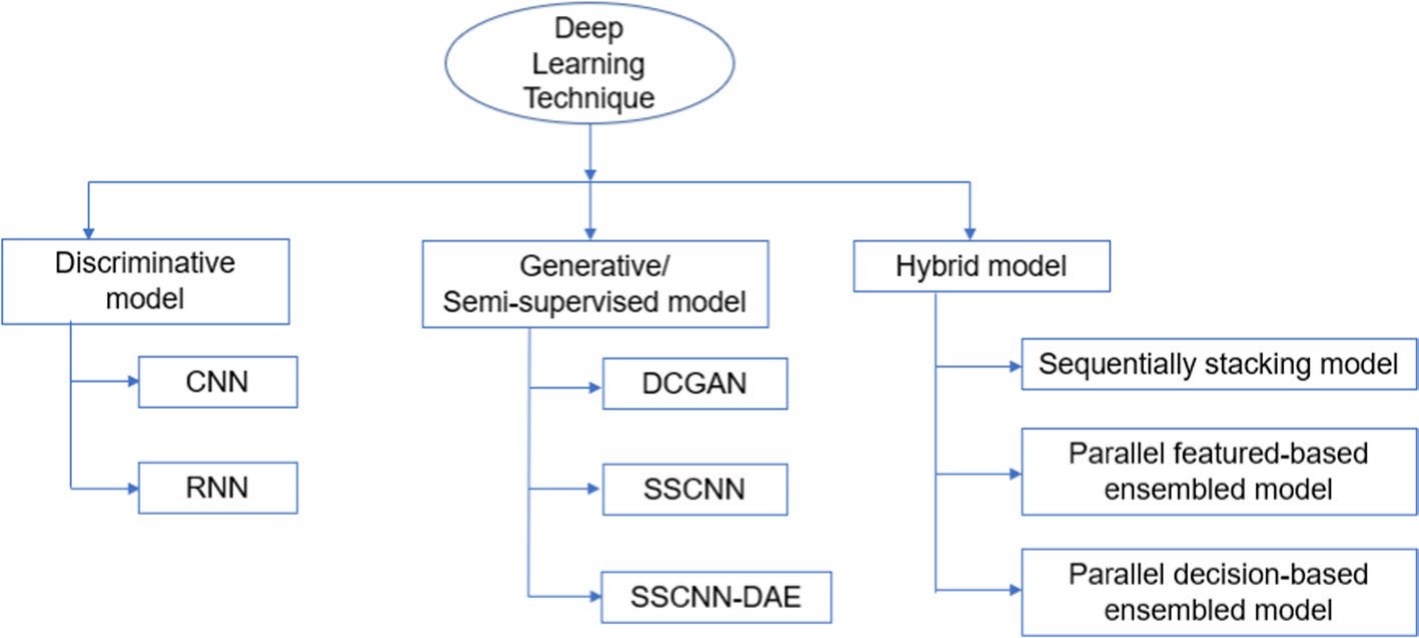
Classification of DL models. CNN: Convolutional Neural Network; RNN: Recurrent Neural Network; LSTM: long short-term memory; DCGAN: Deep Convolutional Generative Adversarial Network; SSCNN: Convolutional Neural Network model with self-learning; SSCNN-DAE: Semi-supervised Convolutional Neural Network model with autoencoder

#### Discriminative models

Discriminative models separate data points into different classes and learn the boundaries using probability estimates and maximum likelihood. Discriminative models are most common in glaucoma and have been extensively used in detection, optic disc/cup, and region of interest (ROI) segmentation.

Convolutional Neural Network (CNN): CNN has been widely used in glaucoma diagnosis based on retinal images such as fundus photograph and OCT images. Chen et al. [97] developed one of the earliest CNN models for glaucoma diagnosis based on fundus images from ORIGA and SCES datasets. The CNN model with five layers obtained the best performance with AUCs of 0.838 and 0.898 based on fundus images from the ORIGA (internal testing) and SCES (independent validation) datasets, respectively. Ahn et al. [100] developed a CNN model to discriminate glaucomatous from normal eyes based on 1542 fundus images. The model outperformed LR and InceptionV3 model with an accuracy of 87.9% and AUC of 0.94 on internal testing set. Norouzifard et al. [102] applied transfer learning based on VGG19 and Inception-ResNet-V2 architectures in identifying glaucoma from 447 fundus photograph and re-tested the model on an independent dataset (HRF with 30 fundus images). They reported that that VGG19 obtained 80.0% accuracy on independent dataset. Masumoto et al. [103] developed a CNN model to classify glaucoma patients into four severity levels based on fundus images from 1399 patients/images. The model obtained AUCs of 0.872, 0.830, 0.864, 0.934 for normal vs all glaucoma, early glaucoma, moderate glaucoma, and severe glaucoma, respectively, on the internal testing set. Fuentes-Hurtado et al. [104] applied DenseNet-201 in classifying 1912 rat OCT images into healthy and pathological using leave-P-out CV (*P* = 15) and obtained an AUC of 0.99. Shibata et al. [105] developed a CNN model using the ResNet architecture based on 1364 glaucomatous and 1768 non-glaucomatous fundus images and tested on independent dataset with 60 glaucomatous eyes and 50 normal eyes. The model obtained an AUC of 0.965 (95% CI 0.935–0.996) that was higher than the performance of ophthalmology residents with an AUC between 0.762 and 0.912 and other models, such as VGG16, RF and SVM. Asaoka et al. [31] developed a six-layer DL model to diagnose early-glaucoma based on 4316 OCT images and obtained an AUC of 0.937 (95% CI 0.906–0.968) based on an independent dataset with 114 patients with glaucoma and 82 normal subjects using a DL transfer model, which was significantly higher than the AUC of 0.631 to 0.862 obtained based on other models (RF and SVM). Using the Youden method, the model attained optimal discrimination with a sensitivity of 82.5% and specificity of 93.9%. Phene et al. [109] developed a CNN model based on Inception-v3 architecture to predict referable GON from ONH features using 86,618 color fundus images and validated the model using three independent datasets, then compared the outcome with glaucoma specialists. For referable GON, they achieved AUCs of 0.945 (0.929–0.960), 0.855 (0.841–0.870), and 0.881 (0.838–0.918) based on the fundus images in the first dataset (with 1205 images), second dataset (with 9642 images), and third dataset (with 346 images), respectively. The model’s AUCs ranged from 0.661 to 0.973 based on glaucomatous ONH features. The CNN model detected referable GON with higher sensitivity than compared with eye care providers.

Al-Aswad et al. [289] evaluated the performance of Pegasus (an AI system based on deep learning) in glaucoma screening based on color fundus photographs by comparing with six ophthalmologists. They found there was no statistically significant distinction between Pegasus (AUC of 0.926, sensitivity and specificity of 83.7% and 88.2%, respectively) and the “optimal” consensus among ophthalmologists (AUC of 0.891, sensitivity and specificity of 61.3–81.6% and 80.0–94.1%, respectively). The correspondence between Pegasus and the gold standard yielded a score of 0.715, whereas the highest level of agreement between ophthalmologists and the gold standard stood at 0.613. Jammal et al. [112] developed a CNN model using ResNet34 architecture (M2M DL) to predict RNFL thickness and grading glaucomatous eyes based on 32,820 pairs of fundus photographs and SD-OCT scans. A total of 490 images were used for testing the model, then the outcome was compared with two glaucoma specialists, the predicted RNFL thickness obtained through M2M DL exhibited a notably stronger absolute correlation with SAP mean deviation (*r =* 0.54) compared to the probability of GON determined by human graders (*r =* 0.48; *P* < 0.001). Furthermore, the M2M DL algorithm demonstrated a significantly higher partial AUC compared to the probability of GON assessed by human graders (partial AUC = 0.529 vs 0.411, respectively; *P* = 0.016). Yu et al. [274] trained a 3D CNN to estimate global VF indices based on macula and optic disc OCT scans from 10,370 eyes, and showed that integrating information from macula and optic disc scan achieved better result compared with inputting separate scan. The combined scan obtained 0.76 Spearman’s correlation coefficient and 0.87 Pearson’s correlation based on VFI and MD while the median absolute error was 2.7 for VFI and 1.57 dB for MD from one of the 8-fold CVs. Wang et al. [34] compared four different CNN models for detecting glaucoma based on RNFL thickness maps from 93 glaucomatous eyes and 128 healthy eyes and found ResNet-18 and a customized CNN architecture called GlaucomaNet had higher performance compared to SVM and KNN. The ResNet-18 architecture obtained the highest accuracy of 90.5%, with sensitivity of 86.0%, specificity of 93.8%, and AUC of 0.906 using 5-fold CV. Kim et al. [113] developed DL models for glaucoma diagnosis based on 1903 fundus images using VGG16 and ResNet-152-M architectures and employed Grad-CAM to visualize regions that were more important for the model to make diagnosis. They used both the whole fundus image as well as cropped versions with OD region only and observed that the ResNet-152-M model achieved an accuracy of 96%, sensitivity of 96%, and specificity of 100% based on 220 fundus images from an independent dataset.

CNN models have been also applied to VFs to detect glaucoma. Kucur et al. [101] proposed an eight-layer CNN model for discrimination of early-glaucoma versus control samples, trained on Glaucoma Center of Semmelweis University in Budapest (BD) dataset with 2267 OCTOPUS G1 VFs (30°) and Rotterdam Eye Hospital (RT) dataset with 2573 HFA VFs. The CNN model had the highest average precision (AP) with 0.874 and 0.986 based on BD and RT datasets, respectively, using 10-fold CV. Performance was similar to other methods using MD thresholds and NN model.

CNN models were also applied to ROI localization in glaucoma studies. Mitra et al. [108] developed a CNN mode to detect the bounding box coordinates of OD that acts as a ROI based on fundus images from MESSIDOR and Kaggle datasets and tested the model using fundus images in the DRIVE and STARE datasets. The average IOU of their model was 96.83%, 95.45%, 96.19%, 95.93% on internal testing set of MESSIDOR and Kaggle, independent datasets of DRIVE and STARE, respectively.

CNN models have been widely employed in optic disc/cup segmentation, which plays an important role in glaucoma detection as CDR is a glaucoma risk factor. Kim et al. [114] developed a fully convolutional networks (FCN) with U-Net architectures for optic disc/cup segmentation based on fundus ROI region from 750 fundus images of RIGA dataset. The best segmentation results for OD showed Jaccard index of 0.95, F-measure of 0.98, and accuracy of 99%. The best segmentation results for OC showed Jaccard index of 0.80, F-measure of 0.88, and accuracy of 99% evaluated by 5-fold CV. Xie et al. [210] developed a new method to segment inner retina thickness using 41 OCT macular scans. The approach addressed spike-like segmentation errors and lack of contextual data by reconstructing more B-scans, concatenating smoothed and contrast-enhanced images into a six-channel input image stack, and merging predicted surfaces from both horizontal and vertical B-scans. The suggested method surpassed the performance of cutting-edge techniques when it came to mean absolute surface distances (normal: 2.18, glaucoma: 3.02), Dice coefficients (GCIPL normal: 0.952, GCIPL glaucoma: 0.899), and Hausdorff distance (RNFL-GCL normal: 12.1; RNFL-GCL glaucoma: 28.9) in an independent test dataset. Li et al. [182] developed a joint OD and OC segmentation model using a region-based DCNN (R-DCNN) based on 2440 fundus images and validated the model based on both in-house testing dataset and public datasets (DRISHIT-GS and RIM-ONE v3) and compared with that of ophthalmologists. The model achieved high Dice similarity coefficient (DC) and Jaccard coefficient (JC) for both OD (DC: 98.51%, JC: 97.07%) and OC (DC: 97.63%, JC: 95.39%) segmentation on the in-house dataset, comparable to that of ophthalmologists. On DRISHTI-GS and RIM-ONE v3 datasets, the model obtained higher DC (DRISHTI-GS: OD: 97. 23%, OC: 94.56%; RIM-ONE v3: OD: 96.89%, OC: 88.94%) and JC (DRISHTI-GS: OD: 94.17%, OC: 89.92%; RIM-ONE v3: OD: 91.32%, OC: 78.21%) values than previous studies.

An automatic two-stage glaucoma screening system was developed by Sreng et al. [291] and was evaluated on 2787 retinal images from 5 public datasets (REFUGE, ACRIMA, ORIGA, RIM-ONE and DRISHTI-GS1). The system utilized DeepLabv3 + combined with pretrained networks for OD segmentation in the first stage and pretrained networks ensembled with SVM for glaucoma classification in the second stage. The best model for OD segmentation achieved high accuracy (99.70%), Dice coefficient (91.73%), and IoU (84.89%) on REFUGE dataset based on the combination of DeepLabv3 + and MobileNet. The ensembled classification model outperformed conventional methods with high accuracy (97.37%, 90.00%, 86.84%, 99.53% and 95.59%) and AUC (100%, 92.06%, 91.67%, 99.98% and 95.10%) values on RIM-ONE, ORIGA, DRISHTI-GS1, ACRIMA and REFUGE datasets.

Recurrent Neural Network (RNN): RNN is specifically designed to handle sequential data by preserving an internal state, enabling the network to remember information from prior inputs. The network takes a sequence of inputs, one at a time, and updates its internal state based on the current input and its previous state. The output of the network at each step depends on the current input and the current state. Long Short-Term Memory (LSTM) networks were introduced as a specialized type of RNN. LSTM networks incorporate memory cells and forget gates, allowing them to manage information as it enters and exits the memory, thus mitigating the drawbacks of traditional RNNs. Veena et al. [145] used an RNN-LSTM model with three dense and three dropout layers and one batch normalization layer for glaucoma diagnosis based on the segmentation result of the fundus images from DRISHTI-GS (101 images) database (no report of classification accuracy). LSTM has been successfully applied to longitudinal data in glaucoma studies as well. Dixit et al. [147] used LSTM to assess glaucoma progression based on longitudinal VF data from 11,242 eyes. Using four consecutive VFs for each subject, the convolutional LSTM network achieved an accuracy of 91–93% when evaluated against various conventional glaucoma progression algorithms. The model trained on both VFs and clinical data displayed superior diagnostic capabilities (AUC:0.89–0.93) compared to a model exclusively trained on VF (AUC:0.79–0.82, *P* < 0.001) using 3-fold CV. In summary, the majority of the studies using discriminative DL models in glaucoma have applied transfer learning and compared performance of different CNN model architectures in a specific task, such as classification based on fundus, VFs, or OCT images. Among the pretrained CNN architectures in glaucoma studies, ResNet has been the most popular architecture used in CNN models and has achieved a higher accuracy compared to the other CNN architectures.

#### Generative Adversarial Network (GAN)/semi-supervised model

Generative Adversarial Networks (GANs) represent a recent breakthrough in DL. First proposed by Goodfellow et al. [292], it constitutes two networks one for image generation and the other for discrimination (between the generated image and authentic). This model has demonstrated high levels of performance in a variety of applications including glaucoma to generate synthesized retinal images in a semi-supervised learning fashion. Diaz-Pinto et al. [240] developed a new retinal image synthesizer and a semi-supervised learning approach for glaucoma assessment utilizing a Deep Convolutional Generative Adversarial Network (DCGAN) based on a dataset consisting of 86,926 fundus images. The model was able to generate (close to) realistic retinal images and discriminate glaucomatous eyes from normal ones with an AUC of 0.9017, specificity of 79.86%, sensitivity of 82.90%, and F1-score of 0.8429 based on the internal testing set. Tang et al. [205] developed a semi-supervised model using a multi-level amplification iterative training method to detect glaucoma based on three different datasets; Sanyuan dataset (11,443 images), Tongren dataset (7806 images), and Xiehe dataset (4363 images). They tested the model based on REFUGE dataset and obtained an accuracy of 95.75%, sensitivity of 87.5%, specificity of 96.7%, and F1-score of 0.919, which were higher than the accuracy of the models previously published. Alghamdi et al. [241] developed a semi-supervised CNN model with self-learning (SSCNN) and Semi-supervised CNN model with autoencoder (SSCNN-DAE) based on both labeled and unlabeled data from RIM-ONE and RIGA datasets. Compared with transfer CNN (TCNN), SSCNN-DAE obtained a higher accuracy of 93.8%, sensitivity of 98.9% and AUC of 0.95 based on the internal testing set.

Overall, GAN and semi-supervised learning can improve the model performance significantly compared to supervised DL models when dealing with small datasets or datasets with limited number of labeled images. Because this is a typical problem in glaucoma studies, such models may be applicable to address related glaucoma challenges. However, GAN has been not widely used in glaucoma studies probably because there is a huge concern related to synthesizing retinal images in ophthalmology [293].

#### Hybrid models

Hybrid models, or fusion networks, are formed based on combining multiple DL models using a single modality or multiple modalities. It has demonstrated a better performance in some glaucoma diagnosis scenarios than a single CNN model [294–298]. In addition, Muhammad et al. [107] developed a hybrid deep CNN model based on AlexNet architecture to extract features from OCT scans, coupled with RF classifier to distinguish healthy suspects and mild glaucoma using 102 eyes. The model with the input of RNFL probability map had the best accuracy of 93.1% using leave-one-out validation. An et al. [110] developed a hybrid CNN mode based on fundus and OCT scans from 208 glaucomatous eyes and 149 healthy eyes. They first trained a VGG19 architecture separately based on fundus images cantered at optic disc, disc RNFL thickness maps, macular GCC thickness maps, disc RNFL deviation maps, and macular GCC deviation maps, then combined the feature vector representation of each CNN model and used a RF classifier to combine models for glaucoma diagnosis. The hybrid model achieved an AUC of 0.963 based on 10-fold CV. Patil et al. [111] developed GlaucoNet which stacked an autoencoder with a CNN model for glaucoma diagnosis based on fundus images from DRISHTI-GS and DRION-DB datasets. The accuracy, precision, F1-score, recall, specificity, and AUC of the model based on the DRISHTI-GS (internal testing set) were 98.2%, 94.6%, 0.979, 99.6%, 94.6%, and 0.94, respectively. Based on the DRION-DB (internal testing set), the corresponding performance metrics were 96.3%, 93.9%, 0.941, 94.2%, 92.6%, and 0.90, respectively. The model outperformed the previous state-of-the-art studies. Cho et al. [247] developed a hybrid model composed of an ensemble of 56 CNNs with different architectures by averaging the outcome of those models based on 3460 fundus photographs to identify unaffected controls, early-stage, and late-stage glaucoma. The proposed hybrid model demonstrated a significantly better performance compared with the best single CNN model, with an accuracy of 88.1% and an average AUC of 0.975 based on 10-fold CV. Akbar et al. [248] developed a hybrid model by combining the DenseNet and DarkNet CNN architectures for glaucoma diagnosis based on 1270 fundus images from HRF, RIM 1, and ACRIMA databases. The hybrid model outperformed the two single CNN networks and achieved an accuracy of 99.7%, sensitivity of 98.9%, and specificity of 100% based on the HRF as the internal validation set. Based on the RIM1 as the internal validation set, accuracy, sensitivity, and specificity were 89.3%, 93.3%, 88.46%, respectively, while based on ACRIMA as the internal validation set, accuracy, sensitivity, and specificity were 99.0%, 100%, and 99%, respectively. Joshi et al. [178] developed a hybrid model based on the ensemble of VGGNet-16, ResNet-50, and GoogLeNet CNN architectures using fundus images collected from a private dataset (PSGIMSR with 1150 images) and three publicly available datasets (DRISHTI-GS with 101 images; DRIONS-DB with 110 images; and HRF with 30 images). The hybrid model outcome was formed based on the majority voting of the three models. The hybrid model yielded an accuracy of 91.13%, sensitivity of 86.58%, and specificity of 95.21% on PSGIMSR dataset, and achieved accuracies of 95.63%, 98.67%, 95.64%, and 88.96%, respectively, on the DRIONS-DB, HRF, DRISHTI-GS, and combined datasets based on 10-fold CV.

It is becoming evident that hybrid CNN models with fusion of different single CNN architectures using a single data modality or multi-modality are being increasingly applied and receiving more attention from investigators. Because glaucoma is a complex and multifactorial disease, hybrid models that utilize different data modalities to detect glaucoma may provide different pieces of information regarding glaucoma to better portray the disease. This is true based on information theory. In addition, multiple sources of information may increase the overall information about the disease. Thus, we predict attention to, and utilization of, hybrid CNN models will continue to increase in glaucoma studies.

Overall, diagnosis, screening of glaucoma and glaucoma progression detection are the major goals in studies discussing various applications of AI in glaucoma. Currently, most of the studies are focused on glaucoma diagnosis or screening and have applied both conventional ML and DL approaches. However, detecting glaucoma progression remains a significant hurdle in clinical practice since detecting true changes due to the disease is challenging. Some of the imaging modalities generate substantial test–retest variability, which complicates distinction between genuine change and fluctuations. Furthermore, there is a lack of consensus regarding specific criteria for glaucoma progression based on VF or structural parameters. Despite these obstacles, several research groups have proposed models for detecting glaucoma progression using traditional ML based on VF test [46, 82, 299] or both VF test and OCT parameters [29, 38]. Wang et al. [299] applied archetypal analysis to detect VF progression from 11,817 eyes and validated it on 397 eyes and achieved an agreement (kappa) and accuracy of 0.51 and 77%, respectively. They showed that archetypal analysis was more accurate than Advanced Glaucoma Intervention Study (AGIS) scoring, Collaborative Initial Glaucoma Treatment Study (CIGTS) scoring, MD slope, and permutation of pointwise linear regression (PoPLR). Shuldiner et al. [46] assessed several ML models to predict rapid progression based on 22,925 initial VFs from 14,217 patients and found SVM as the best performing model with an AUC of 0.72 (95% CI 0.70–0.75). Lee et al. [38] included 33 initial clinical parameters in ML models to predict normal-tension glaucoma (NTG) progression in young myopic patients based on 155 patients and obtained an AUC of 0.881 (95% CI 0.814–0.945) based on extremely randomized tree which was better than RF. There are also several studies that applied DL models to detect or predict progression based on VF and clinical data [147], OCT images [237, 243] or fundus images [164]. Bowd et al. [243] developed a DL-autoencoder (AE) to detect glaucoma progression based on OCT RNFLROI from 44 progressing, 189 non-progressing, and 109 healthy eyes. The DL-AE ROIs achieved sensitivity and specificity of 0.90 and 0.92, respectively, which was higher than models based on global cpRNFL annulus thicknesses. Li et al. [164] developed AI models to predict glaucoma progression based on color fundus photographs and demonstrated AUCs of 0.87 (0.81–0.92) and 0.88 (0.83–0.94) based on two external datasets. Successful detection of progression may facilitate earlier interventions thus diminishing the likelihood of patients experiencing vision impairment due to glaucoma over their lifetimes. Nevertheless, existing studies have not showed solid results in forecasting the rate of progression or the timing of its occurrence.

## Discussion

AI is an active research area that encompasses a wide range of approaches of ML, employing different applications such as ML and DL algorithms, which have been successfully applied in various domains such as image processing, pattern recognition, speech recognition, and natural language processing. When applied in medical fields such as ophthalmology, these models show promising potential for improving access to health care and enhancing patient outcomes. Early ML models in the form of neural networks were applied to VFs for diagnosing glaucoma in the 1990’s [300, 301]. With later advancements in AI models, various groups demonstrated the efficacy of these models in detecting glaucoma. The following is a summary of our findings regarding AI models in glaucoma. DL-based models are typically more accurate than conventional ML approaches, as evidenced by their performance in glaucoma applications such as image processing, pattern recognition, diagnosis, and prognosis. ML/DL techniques, such as CNNs, RNNs, LSTM, GAN, DBN, etc., can be easily adapted to various glaucoma problems including screening, diagnosis, and prognosis. DL-based techniques can handle more complicated problems such as high-dimensional data and interactions between different data modalities. Ensemble DL models can improve the performance of glaucoma detection. Combination of multiple modalities of the data can improve the model performance. However, there are also several limitations with respect to AI liability, reimbursement, and ethical principles, including non-maleficence of AI models in glaucoma, patient autonomy, and equity and absence of bias in benefits and rights, that are out of the scope of this article and may be discussed in more focused future studies.

### Datasets

Datasets serve as the key element for developing AI models and they play a critical role in reproducibility and generalizability. Conventional statistical learning models can be applied to clinical datasets to identify associations even based on a relatively small sample size. However, in addition to quality, quantity is also important in developing emerging DL models. For instance, labeling fundus images by non-glaucoma experts may degrade the quality of the dataset, leading to non-solid constructs. Increased quantity, along with improved quality, usually leads to better model performance and higher likelihood of generalizability. Well-annotated, multi-modal datasets can also positively impact AI models for more accurate detection of the disease. However, obtaining a sufficiently large dataset with several modalities can be challenging due to numerous hurdles such as low disease prevalence, data confidentiality, data protection regulations, and labor-intensiveness of the process.

Moreover, lack of standardized definitions for glaucoma poses another challenge in achieving consistent evaluation of the AI models. Christopher et al. [136] investigated the impact of study population, labeling, and training on glaucoma detection using AI models, and found that the diagnosis performance varied based on the reference standard (RS) and labeling strategy. To develop solid AI models for detecting glaucoma, it is vital to train the models based on dependable datasets and evaluate the models based on consistent ground truths. Thakoor et al. [281] attempted to improve the generalizability of AI models in glaucoma detection and found that the model trained and tested with the same RS demonstrated the highest accuracy. However, substantial disagreement, even among experienced glaucoma specialists, makes it challenging to establish a uniform reference for ground-truth labeling [281].

Many of the currently available datasets have been gathered from populations with limited representation of different ethnicities, used specific hardware and imaging settings to collect data, and used high-quality images that are far from real-world settings. One potential drawback of lack of diversity within the models, is that these datasets may not readily generalize to real-world patient populations. Asaoka et al. [118] applied ResNet model based on 3132 fundus images 0.877 to 0.948 and from 0.945 to 0.997 based on two independent validations datasets. Nonetheless, their model was not evaluated based on diverse ethnic groups, which is important as fundus images from patients with different ethnic backgrounds may exhibit distinct characteristics such as variation in retina color as well as optic disc structure.

### AI models

Although there are several different CNN-based AI models reported in the literature with high performance in detecting glaucoma, the generalizability of those models is questionable since independent validation is lacking in many studies. Independent validation is a crucial step in developing AI models because it can determine whether a model has learned meaningful disease-related features and patterns to generalize well to unseen data. It also aids uncovering potential biases or overfitting issues when the model performs exceptionally well based on the training data but performs poorly based on the new data. Moreover, assessing the robustness of AI models against variations and changes in the data distribution is especially important when deploying models in real-world applications where input data may change over time. Additionally, validating AI models based on independent (accessible) datasets allows for fair comparison of AI models using the same evaluation dataset, ensuring transparency. For the literature we reviewed, most of the articles only used internal testing or CV, and less than about one-third of the articles used independent validation, which are summarized in Table 1. Also, many models were developed based on very clean datasets, for example including only high-quality images and clear pathological features, whereas this does not reflect the case in real-world data. In addition, many models demonstrated good performance in differentiating healthy versus moderate/severe glaucoma; however, this does not add significant clinical value, as differentiation between these two types of conditions is also easily achieved by clinicians. Thus, more helpful models should be developed based on a wide spectrum of glaucoma severity subjects, including early-stage glaucoma.

**Table 1:** Summary of studies that have used independent validation or compared the model against human experts

References	Task	Output label	Data modality	No. of sample for model development	No. of sample for independent validation	No. of human expert	Model used	Key results	Strengths/limitations
Nawaz et al. [166]	Glaucoma detection	Healthy, glaucoma	Fundus	ORIGA (650 images)	HRF (30 images), RIM ONE (485 images)		Bi-directional Feature Pyramid Network (BiFPN) EfficientDet-D0 (EfficientNet-B0 as backbone)	Train on ORIGA, test accuracy: HRF-98.21%; RIM ONE–97.96%; train on ROM-ONE, test accuracy: ORIGA–97.83%, HRF–98.19%.	Pros: high performance. Cons: validation dataset is small.
Gong et al. [249]	Glaucoma diagnosis	Normal, glaucoma	Fundus		1000 images	4 doctors	Hierarchical structure (HDLS) AI system + SVM	Doctors’ performance was improved with the assistance of AI. Overall doctors' performance without AI assistance (round1): sensitivity: 65%, specificity: 78%; accuracy: 71.5%; Overall doctors' performance with AI assistance (round 2): sensitivity: 91%, specificity: 88%; accuracy: 89.5%.	Pros: AI and human comparison. Cons: small number of tested samples (200 each round) and number of experts; self-learning effect exists.
Yugha et al. [239]	Glaucoma detection	Healthy, glaucoma	Fundus	ORIGA (650 images)	RIM-1-DL (485 images); HRF (45 images)		Bi-Directional Feature Pyramid system modules of EfficientDet-DO (EfficientNet-B0 backbone)	Accuracy: HRF: 97.89%; RIM 1: 97.64%.	Pros: high performance. Cons: validation dataset is small.
Ko et al. [287]	Glaucoma detection	Non-glaucoma, glaucoma	Fundus	TVGH (944 images)	CHGH (158 images); DRISHTI-GS1 (101 images); RIM-ONE r2 (455 images)		EfficientNet B3	CHGH: AUC: 0.910 (0.798–1.000); accuracy: 80%; sensitivity: 65%; specificity: 95%; RIM-ONE r2: AUC: 0.624 (0.501–0.748); accuracy: 52.5%; sensitivity: 15%; specificity: 90%; DRISHTI-GS1: AUC: 0.770 (0.558–0.982); accuracy: 55%; sensitivity: 10%; specificity: 100%.	Pros: validated on multiple datasets. Cons: performance was not generalizable in RIM-ONE r2 and DRISHTI-GS1.
Xue et al. [228]	Glaucoma detection; severity classification	Normal, mild, moderate, severe	IOP, fundus, VF	6131 samples	240 samples	8 juniors,3 seniors,3 experts	Multi- feature deep learning (MFDL) (DetectionNet, ClassificationNet) (ResNet backbone)	MFDL achieved a higher accuracy of 0.842 (95% CI, 0.795–0.888) than the direct four classification deep learning (DFC-DL, accuracy of 0.513 [0.449–0.576]), CFP-based single-feature deep learning (CFP-DL, accuracy of 0.483 [0.420–0.547]) and VF-based single-feature deep learning (VF-DL, accuracy of 0.725 [0.668–0.782]) Its performance outperformed 8 juniors,3 seniors and 1 expert and was comparable with 2 glaucoma experts	Pros: compared with human expert. Cons: validation dataset is small and not from multi-center.
Wu et al. [47]	Glaucoma screening; subtyping; early diagnosis	Screening (glaucoma, healthy); subtyping (POAG, PAAG); early POAG	Tear metabolic fingerprinting (TMF)	266 samples	54 samples		Ridge regression (RR)	Identified metabolic biomarker (Lac, Thr, Mer, Sul, Bar, or DPAE) for glaucoma characterization Glaucoma Screening AUC: 0.856 (95% CI: 0.757–0.954).	Pros: biomarkers were identified; simple model with good performance. Cons: mass spectrometer is essential for the data; larger dataset is needed for further validation.
Singh et al. [173]	Glaucoma diagnosis	Normal, glaucoma	Fundus	ACRIMA(705 mages), ORIGA (650 images), HRF (30 images)	DRISHTI-GS (101 images); PRIVATE (33 images)		InceptionResNet-V2	AUC: 0.9042; accuracy: 90%; sensitivity: 86.748%; specificity: 94.11%; F1-score: 91.13%.	Pros: compared multiple models. Cons: small validation and training dataset.
Noury et al. [175]	Glaucoma diagnosis; severity classification	Normal, glaucoma; mild, moderate severe	SD-OCT ONH scans	2461 OCT scans volumes	Hong Kong (HK): 1625 scans; India: 672 scans; Nepal: 380 scans	1	DiagFind: 3D CNN	AUC: HK: 0.80 (95% CI, 0.78–0.82), India:0.94 (95% CI, 0.93–0.96), Nepal: 0.87 (95% CI, 0.85–0.90); sensitivity: HK: 0.73 (0.67–0.79); India: 0.93 (0.88–0.99); Nepa: 0.79 (0.68–0.90); specificity: HK: 0.73 (0.61–0.85); India: 0.71 (0.51–0.91); Nepa: 0.79 (0.66–0.92); F1-score: HK: 0.76 (0.75–0.77); India: 0.91 (0.90–0.92); Nepal: 0.80 (0.78–0.83) testing set from Stanford (100 cases): AUC: 0.92 (95% CI, 0.90–0.93), human grader: 0.91	Pros: validated result on rea-world datasets from multiple sites. Cons: exclude cases without consensus labeling and difficulty to be diagnosed by skilled clinicians.
Fan et al. [176]	Glaucoma diagnosis	Healthy, glaucoma	Fundus	OHTS: 66,715 images	ACRIMA (705 images); LAG (4854 images); DIGS (9473 images)		ResNet-50	AUC: DIGS: 0.74 (0.69–0.79); ACRIMA: 0.74 (0.70–0.77); LAG: 0.79 (0.78–0.81); Sensitivity (at 85% specificity): DIGS: 0.52; ACRIMA: 0.46; LAG: 0.59; Sensitivity (at 95% specificity): DIGS: 0.30; ACRIMA: 0.29; LAG: 0.42	Pros: validated results on multiple external datasets. Cons: the model was not generalizable in external datasets.
Fan et al. [222]	Glaucoma diagnosis	Healthy, glaucoma	Fundus	OHTS: 66,715 images	DIGS (10,473 images), ACRIMA (705 images), LAG (4854 images), RIM-ONE (455 images), ORIGA (650 images)		Data-efficient image Transformer (DeiT)	AUC: OHTS: 0.91 (0.87, 0.93), DIGS:0.77 (0.71, 0.82); ACRIMA: 0.740.74 (0.70, 0.77); LAG: 0.88 (0.87, 0.89); RIM-ONE:0.91 (0.88, 0.94); ORIGA: 0.73 (0.68, 0.77); sensitivity (at 85% specificity): OHTS:0.79; DIGS: 0.57; ACRIMA: 0.46; LAG: 0.77; RIM-ONE: 0.83; ORIGA: 0.40; sensitivity (at 95% specificity): OHTS:0.56; DIGS: 0.34; ACRIMA: 0.31; LAG: 0.59; RIM-ONE: 0.73; ORIGA: 0.21.	Pros: validated in multiple external datasets; Vision Transformers have the potential to improve generalizability. Cons: cropped images may lose information.
Huang et al. [231]	Glaucoma diagnosis	Normal, glaucoma	Fundus, VF	1655 samples	196 samples		Probabilistic deep learning model (EffientientNetB4 backbone)	AUC: 0.98 (0.98–0.99); accuracy: 93% (92–95%); sensitivity: 91% (87–95%); specificity: 95% (94–96%).	Pros: quantifying the uncertainty of the model Cons: dataset was from COMPASS instrument; more external validation is needed.
Huang et al. [146]	Glaucoma VF grading	Clear, mild, moderate, severe, diffuse	VF (HFA and Octopus)	3805 VFs (Octopus); 13,231 VFs (HFA)	150 VFs (HFA)	2 ophthalmic clinicians, 6 medical students	Fine-grained grading deep learning system (FGGDL: FGG-O, FGG-H); Interactive Interface	AI outperformed human experts and their performance was improved with the assistance of AI AUC: FGGDL: 0.893 (0.862–0.923); clinician 1: 0.838 (0.801–0.874); clinician 2: 0.833 (0.796–0.869); all the other medical students' performance was lower than 0.80.	Pros: external validation is applied and compared with human experts. Cons: high test–retest variability was not considered.
Li et al. [182]	OD/OC segmentation, glaucoma screening	Glaucoma, non-glaucoma	Fundus	In-house (2440 images)	DRISHIT-GS (101 images); RIM-ONE v3 (159 images)	4 ophthalmologists	R-DCNN (DAC-ResNet34)	The segmentation results of in-house testing set were comparable to that of human experts', OD: DC-98.51%, JC-97.07%; OC: DC-97.63%, JC-95.39% Segmentation results of DRISHTI-GS and RIM-ONE v3 are better than existing studies: DRISHTI-GS: OD: DC-97.23%, JC-94.17%; OD: DC-94.56%, JC-89.92%. RIM-ONE v3: OD: DC-96.89%, JC-91.32%; OC: DC-88.94%, JC-78.21%. glaucoma screening: AUC: DRISHTI-GS:0.968; RIM-ONE v3:0.941.	Pros: compared with human expert and existing studies. Cons: small sample size of training and validation dataset.
Li et al. [164]	Glaucoma diagnosis, glaucoma incidence/progression prediction	Diagnosis (glaucoma, non-glaucoma); glaucoma incidence prediction: with/without glaucoma development; glaucoma progression prediction: with/without progression	Fundus	**Diagnosis**: 24,054 eyes; **predict glaucoma incidence**: 11,548 eyes; **predict glaucoma progression:** 3425 eyes	Glaucoma diagnosis: external test 1: 6162 images (eyes), external test 2: 824 images (eyes); glaucoma incidence: external test 1: 955 images, external test 2: 719 images; glaucoma progression: external test 1: 337 images, external test 2: 513 images		DiagnoseNet, PredictNet	Glaucoma diagnosis: AUC: test 1: 0.94 (0.93–0.94); test 2–0.91 (0.89–0.93), sensitivity: glaucoma diagnosis: test 1:0.89(0.87–0.90); test 2: 0.92 (0.88–0.96), specificity: test 1: 0.83 (0.81–0.84); test 2: 0.71 (0.67–0.74); Predict glaucoma incidence: AUC: test 1: 0.89 (0.83–0.95); test 2: 0.88 (0.79–0.97); sensitivity: test 1: 0.84 (0.81–0.86); test 2: 0.84 (0.81–0.86); specificity: test 1: 0.68 (0.43–0.87); test 2: 0.80 (0.44–0.97); Predict glaucoma progression: AUC: test 1: 0.87 (0.81–0.92) and test 2: 0.88 (0.83–0.94); sensitivity: test 1: 0.82 (0.78–0.87) and test 2: 0.81 (0.77–0.84); specificity: test 1: 0.59 (0.39–0.76) and test 2: 0.74 (0.55–0.88).	Pros: performed multiple tasks (diagnosis, incidence and progression prediction) and include multiple external validation. Cons: exclude low quality images, validation dataset were only Chinese datasets.
Mehta et al. [149]	Glaucoma detection	Healthy, glaucoma, PTG (progress to glaucoma)	Demographic, systemic and ocular data, color fundus, OCT	UK Biobank (2574 eyes, glaucoma-1193 eyes, healthy-1283 eyes, PTG-98 eyes)	200 eyes	5 glaucoma-fellowship trained ophthalmologists	InceptionResnetV4	Best model with input of OCT, color fundus, systemic and ocular data obtained AUC = 0.967 (95% CI 0.93–1.0); Human expert (only made diagnosis on color fundus): AUC: 0.79–0.84.	Pros: used multiple modalities, several methods for interpret DL model (SHAP, saliency map); Cons: poor quality of the fundus images may contribute low AUC.
Hemelings et al. [151]	Glaucoma detection, VCDR regression	Glaucoma, non-glaucoma	Fundus	UZL (13,551 images)	REFUGE (1200 images)		ResNet50	AUC: original fundus: 0.87(95% CI 0.83–0.91); 60% ONH cropping: 0.80 (95% CI 0.76–0.84).	Pros: report explainability analysis by cropping ONH area; Cons: applying masks of fixed size might lead to a small variation in visible features across fundus images due to variation in ONH size across the study population.
Thakoor et al. [279]	Glaucoma detection	Glaucoma, non-glaucoma	OCT images (RNFL probability maps)	737 eyes	135 eyes	2 expert OCT readers	InceptionV3 + FC (with concept activation vectors (TCAVs))	The TCAVs scores were consistent with features used by human experts based on eye fixations AUC:0.911.	Pros: applied test with concept activation vectors (TCAVs) for model interpretability; Cons: multi-center datasets were not in external validation.
Thakoor et al. [281]	Glaucoma detection	Glaucoma, non-glaucoma	OCT B-Scans, RNFL probability maps	RNFL maps: 737 eyes; B-scans: 771 eyes	RNFL maps:135 eyes; B-scans: 125 eyes		CNN A (ResNet18 + RF) with RNFL-map as input	CNN generalizability can be improved with data augmentation, multiple input image modalities, and training on images with confident ratings. choosing a thorough and consistent RS for training and testing improves generalization to new datasets Best result was with RNFL map input and data augmentation on CNN A: AUC = 0.918 (95% CI 0.866–0.970), accuracy = 85.9%;	Pros: improved generalizability by several techniques (multi-modalities, consistent labels, data augmentation); Cons: independent validation can be improved by a larger dataset.
Natarajan et al. [143]	Glaucoma detection, OD segmentation	Glaucoma, normal	Fundus	RIGA (750 images), RIM-ONEv2 (455 images)	ACRIMA (705 images), Drishti-GS1 (101 images), RIMONEv1 (169 images)		UNet-Snet (SqueezeNet)	glaucoma detection: AUC: ACRIMA: 100%; Drishti-GS1: 99.90%, RIMONEv1: 100%; accuracy: ACRIMA: 99.86%; Drishti-GS1: 97.05%, RIMONEv1: 100%; sensitivity: ACRIMA: 100%; Drishti-GS1: 100%, RIMONEv1: 100%; specificity: ACRIMA: 99.75%; Drishti-GS1: 90.32%, RIMONEv1: 100%.	Pros: achieved high performance; Cons: small dataset for training and independent validation.
Kenichi et al. [129]	Glaucoma diagnosis	Glaucoma, normal	Fundus	3132 images (from ordinary camera)	162 images (from ordinary camera and smartphone)		ResNet34	AUC: camera-98.9%; smartphone-84.2% advanced glaucoma: AUC: camera-99.3%; smartphone-90.0%.	Pros: validated performance between fundus from ordinary camera and smartphone; Cons: training images were all from ordinary camera, poor image quality of fundus from smartphone.
Xu et al. [242]	Glaucoma diagnosis	Referable glaucomatous optic neuropathy (GON), unlikely GON	Fundus	1791 images	dataset1: 6301 images dataset2: 1964 images dataset3: 400 images	12 (4 senior ophthalmologists, 4 junior ophthalmologists, and 4 technicians)	Hierarchical deep learning system (HDLS)(segmentation- classification, Inception-v3 backbone)	Reliable region has higher sensitivity and specificity than suspicious region datset1: AUC = 0.981 (95% CI, 0.978–0.985), reliable region: sensitivity = 97.7% (95% CI, 97.0–98.3%), specificity = 97.8% (95% CI, 97.2–98.4%); dataset2: AUC = 0.983 (95% CI, 0.977–0.989); reliable region: sensitivity: 98.4% (95% CI, 97.3–99.5%), specificity = 98.2% (95% CI, 97.4–99.1%) Dataset 3: performance of human experts were improved with the referring of HDLS: senior group: sensitivity: 0.93 (diagnose independently), 0.96 (referring to HDLS); specificity: 0.88 (diagnose independently), 0.95 (referring to HDLS).	Pros: this system is transparent and interpretable. the results were validated on three large validation datasets and results were comparable to that of human experts. Cons: validation dataset did not include data from other ethnics than Chinese.
Bhuiyan et al. [246]	Glaucoma diagnosis	Glaucoma-suspect, not-suspect	Fundus	1546 disc-centered fundus images (AREDS, SiMES, RIM-ONE)	ORIGA (638 gradable images)		Ensemble of Xception, Inception-Resnet-V2, NasNet, Inception-V3	AUC: 0.85; accuracy: 83.54%; sensitivity: 80.11%; specificity: 84.96%.	Pros: screening glaucoma suspect is important; Cons: CDR is not the only biomarker for glaucoma suspect, other biomarkers, such as CDR asymmetry can be added in future studies.
Tang et al. [205]	Glaucoma diagnosis	Glaucoma, non-glaucoma	Fundus	Sanyuan (11,443 images), Tongren (7806 images), Xiehe (4363 images)	REFUGE (1200 images)		AMNet (semi-supervised learning)	accuracy: 95.75%; sensitivity: 87.5%; specificity: 96.7%; F1-score: 91.9%.	Pros: the model boosted the robustness of model with limited labeled data. Cons: diverse validation dataset might be needed to future validate the model.
Alghamdi et al. [241]	Glaucoma diagnosis	Glaucoma, normal	Fundus	RIM-ONE (455 images) RIGA (750 images)		2 ophthalmologists	TCNN-Transfer Convolutional Neural Network (VGG16); SSCNN- Semi-supervised Convolutional Neural Network model with self-learning; SSCNN-DAE-Semi-supervised Convolutional Neural Network model with autoencoder	three deep learning CNN models outperform the performances of both ophthalmologists with clear margins. RIM-ONE: accuracy: two experts attain 59.2% and 55.4.0% SSCNN-DAE: AUC: 0.95, accuracy: 93.8%; sensitivity: 98.90%; specificity: 90.50%.	Pros: compared with human experts and outperformed them. Cons: small datasets were used for training; external validation was not included.
Li et al. [278]	Glaucoma diagnosis in myopia	Glaucoma, non-glaucoma	RNFL profile	2223 eyes	508 eyes		FCN + RBFN (radial basis function network) + RNFL compensation	By applying the RNFL compensation algorithm, the AUC for detecting glaucoma increased from 0.70 to 0.84, from 0.75 to 0.89, from 0.77 to 0.89, and from 0.78 to 0.87 for eyes in the highest 10%, 20%, 30% and any axial length (AL), respectively.	Pros: An RBFN has good generalization, strong tolerance to input noise, and online learning ability made it possible to interpret the patterns to a reliable compensation. Cons: compensation of the RNFL profile was based on data from participants with an age ≥ 50 years, this was not validated in younger participants; the validation dataset included a 1:1 ratio of glaucomatous to non-glaucomatous eyes, this is not the case in read world data.
Chiang et al. [277]	Primary angle closure glaucoma (PACG) detection	POAG, PACG	Goniophotography	32,635 images	1000 images	9 graders	CNN (ResNet-50 backbone)	CNN achieved excellent performance based on single-grader (AUC = 0.969) and consensus (AUC = 0.952) labels. The agreement between the CNN classifier and consensus labels (κ = 0.746) surpassed that of all non-reference human graders (κ = 0.578–0.702).	Pros: the model obtained comparable performance with human graders. Cons: the model has limitation of generalizable to other ethnic groups and goniophotography taken from other devices.
Zhao et al. [196]	Cup-to-disc ratio (CDR) estimation, glaucoma screening	Glaucoma, normal	Fundus	Direct-CSU (934 images)	ORIGA (650 images)		Unsupervised feature representation of fundus image with a CNN (MFPPNet-3 blocks DenseNet) + RF	glaucoma screening: AUC: 0.88 CDR estimation: MAE: 0.0606; coefficient of correlation *r =* 0.68.	Pros: estimated the CDR value more effectively than results obtained from traditional segmentation-based methods; potential to handle unlabeled data. Cons: further validation on diverse data sources would be needed.
Jammal et al. [112]	Fundus predict RNFL; glaucoma detection	Glaucoma, normal	Fundus	32,820 pairs of fundus photos and SD-OCT scans		2 graders	M2M DL (ResNet34 backbone)	DL algorithm outperformed human graders in detecting signs of glaucomatous damage on fundus photographs. glaucoma detection: AUC: DL: 0.801 [95% CI: 0.757, 0.845]; human: 0.775 [95% CI: 0.728, 0.823]; AUPRC: DL: 0.810 [95% CI: 0.765, 0.851]; human: 0.761 [95% CI: 0.703, 0.819] RNFL prediction (DL): MAE: 7.39 um; Pearson’s *r =* 0.832.	Pros: the M2M model is able to provide a quantitative output and outperformed human graders. Cons: used the presence of visual field defects as the gold standard may be biased and influence accuracy.
Wang et al. [142]	Glaucoma detection	Glaucoma, normal	OCT images	HK dataset: 975,400 B-scans (4877 volumns)	Stanford dataset: 246,200 B-scans (1231 volumns)	2 glaucoma experts	2D-ResNet18-SEMT (SEmi-supervised Multi-Task)	Stanford (volumn based): AUC: 0.933, accuracy:86%; F1-score: 0.889; human vs model (HK, volumn based): AUC: 0.977 (DL), 0.918 (human); accuracy: 0.927 (DL), 0.912 (human); F1 score: 0.941 (DL); 0.917 (human).	Pros: semi-supervised learning addressed the miss VF measurement label problem in the training set, the multi-task learning network to explore the relationship between the functional and structural, which was beneficial to accuracy improvement. Cons: the framework at the training stage is not end-to-end, where the hard assignment for VF measurement and the multi-tasking training work in a cascaded manner.
Russakoff et al. [119]	Glaucoma diagnosis	Referable, nonreferable glaucoma	3D-OCT	2805 scans	Hongkong:505 eyes, India:336 eyes		gNet3D (3DCNN)	AUC: HK:0.78; India: 0.95	Pros: first studies to use machine learning in risk stratification; multinational external datasets across geographical and ethnicity distribution from Hong Kong and India. Cons: lack of inclusion of suspects in external datasets; different definitions of glaucoma between development and external dataset.
Zaleska-Żmijewska et al. [121]	Glaucoma diagnosis	Glaucoma, healthy	Fundus, IOP	1687 images	Campaign1:752 images (C1) campaign2: 352 images (C2)		AlexNet	images classifier: accuracy: C1:80%; C2:78%; sensitivity: C1:0.73, C2:0.84; specificity: C1:0.83; C2:0.67; fundus + IOP: accuracy: C1:71%; C2:79%; sensitivity: C1:0.79, C2:0.92; specificity: C1:0.67; C2:0.42.	Pros: include IOP risk factor in model; IOP inclusion improved the sensitivity. Cons: small number of datasets, performance need to be improved.
Zheng et al. [130]	Glaucoma diagnosis	Glaucoma, normal	SD-OCT images (hand-craft features (HCFs), peripapillary RNFL OCT images)	1501 images	104 images		Inception-V3	AUC: 0.990 (0.974, 1.000), accuracy: 0. 990(0.974, 1.000); sensitivity: 0.981 (at 80%, 90% specificity).	Pros: achieved higher sensitivity and specificity compared to traditional HCFs. Cons: external validation test is needed from different centers or OCT devices; most of the glaucoma cases were quite severe and this made classification easier in the current study; used images of Chinese eyes only, the results may not be applicable to other populations.
Li et al. [131]	Glaucoma detection	Glaucoma, non-glaucoma	VF (deviation probability plots (PDPs), numerical pattern deviation plots (NDPs), and numeric displays (NDs))	9022 VFs	phase1: test 1: 200 VFs, test 2:406 VFs, test 3: 507VFs phase 2: 649 VFs	6 ophthalmologists	iGlaucoma (2D-Fusion-CNN (with input of ND + NDP + PDP))	In Phase I, the DLS outperformed all six ophthalmologists in the three test sets (AUC of 0.834–0.877, with a sensitivity of 0.831–0.922 and a specificity of 0.676–0.709); In Phase II, iGlaucoma had 0.99 accuracy in recognizing different patterns in pattern deviation probability plots region, with corresponding AUC, sensitivity and specificity of 0.966 (0.953–0.979), 0.954 (0.930–0.977), and 0.873 (0.838–0.908).	Pros: developed ‘iGlaucoma’, a smartphone application-based deep learning system (DLS); the performance outperformed human expert. Cons: this study is limited to the Chinese population; this DLS only utilizes VF, no other modality included.
Kim et al. [132]	Glaucoma diagnosis	Glaucoma, normal	OCT (RNFL thickness, RNFL deviation maps, GCIPL thickness, GCIPL deviation maps, ocular axial length)	8988 images	1420 images	2 glaucoma specialists	VGG-19	The glaucoma-diagnostic ability was highest when the DL system used the RNFL thickness map alone, among combination sets, use of the RNFL and GCIPL deviation map showed the highest diagnostic ability. It showed detection patterns similar to those of glaucoma specialists External (RNFL + GCIPL deviation map): AUC: 0.985 (95% CI 0.966–0.995), sensitivity (at 90% specificity): 97.2%, sensitivity (at 80% specificity): 98.2%.	Pros: include interpretability and compared with human, with agreement pattern; multiple modalities and wide severity level included. Cons: external validation only include good quality OCT images and only from Asian ethnicity.
Christopher et al. [136]	Glaucoma diagnosis	Glaucoma, normal	Fundus	DIGS/ADAGES: 14,822 images; MRCH: 3132 images	Iinan Dataset: 215 images Hiroshima Dataset: 171 images ACRIMA: 705 images		UCSD (ResNet50) UTokyo (ResNet34)	Utokyo (sequential) obtained highest performance in external datasets: AUC: linan:0.97 (0.94–0.99), Hiroshima: 0.99 (0.99–0.99), ACRIMA: 0.86 (0.83–0.89).	Pros: DIGS/ADAGES and MRCH consisted diverse population, the study computed model performance stratified by disease severity, myopia status, and race. Cons: the datasets have inconsistent glaucoma definition and labeling strategy.
Maadi et al. [194]	OD/OC segmentation, glaucoma detection	GLAUCOMA, non-glaucoma	Fundus	Drishti-GS1: 101 images RIMONE v3: 159 images	REFUGE: 1200 images		Modified U-Net (SE-ResNet50)	OD/OC segmentation: F1-score: OD:0.91; OC:0.79. Diagnosis: AUC: 0.939.	Pros: external validation was used and performed well. Cons: larger training and validation datasets might be needed for further improvement.
Phene et al. [109]	Detect referable GON	Non-referable GON, referable GON	Fundus	88,126 images	A: 1205 images B: 9642 images C: 346 images	10 graders (grade 411 images in validation A dataset)	Inception-v3	The performance of DL was better than human on a subset (411 images) of validation dataset A AUC: A:0.945 (0.929–0.960); B: 0.855 (0.841–0.870); C: 0.881 (0.838–0.918).	Pros: this DL algorithm has higher sensitivity and comparable specificity to eye care providers in detecting referable GON in color fundus images; provides insight into which ONH features drive GON assessment by glaucoma specialists. Cons: diagnosis of glaucoma is not based on ONH appearance alone but also relies on the compilation of risk factors.
Gómez-Valverde et al. [261]	Glaucoma detection	Glaucoma, normal	Fundus	2313 images (RIM-ONE, DRISHTI-GS, ESPERANZA)		4 glaucoma experts	VGG19	human expert (ESPERANZA): specificity = 0.8914, sensitivity = 0.7662; model (train on all 3 dataset): AUC: 0.94; sensitivity: 87.01%; specificity: 89.01%.	Pros: model performance was comparable to human expert. Cons: no independent validation dataset provided.
Asaoka et al. [118]	Glaucoma diagnosis	Glaucoma, normal	Fundus	3132 images (camera: Kowa)	Test 1: 205 images (camera: Kowa) test2: 171 images (camera: Topcon)		ResNet	Data augmentation improved model performance With augmentation: AUC: test 1: 0.948 (0.903–0.968); test 2: 0.997 (0.994–1); Without augmentation: AUC: test 1:0.877 (0.828–0.926); test 2: 0.945(0.913–0.976).	Pros: model was validated using images obtained from different fundus cameras and it had a high diagnostic ability irrespective of the type of fundus camera. Cons: the model has not been validated in different ethnicities.
Kim et al. [113]	Glaucoma diagnosis and localization	Glaucoma, normal	Fundus	2123 images	RIM-ONE r3 (159 images)		ResNet152	accuracy: 93.5%; sensitivity: 92.9%, specificity: 92.9%.	Pros: a web application was developed with output pd Grad-CAM. Cons: larger datasets from different instrument may be incorporated to improve the performance.
Asaoka et al. [31]	Diagnose early glaucoma	Glaucoma, normal	Macula SD-OCT (RNFL and GCC thicknesses)	Pretrain: 4316 OCT images train: 178 eyes (94 POAG, 84 normal)	196 eyes (114 POAG, 82 normal)		DL transform (CNN using 8*8 grid RNFL (in the first channel) and GCC data (in the second channel), use both pretraining and training)	With pretraining: AUC: 93.7% (90.6–96.8). Optimum discrimination at sensitivity of 82.5%, specificity of 93.9%. Specificity (at 80% sensitivity): 83.3%; Specificity (at 90% sensitivity): 86.6% Without pretraining: AUC: 76.6–78.8%	Pros: the study tested importance of pretraining process in model performance improvement Cons: the results were obtained using a homogeneous patient population (only Japanese patients), further study might prepare OCT data from multiple ethnicities, to generalize the results
Al-Aswad et al. [289]	Glaucoma diagnosis	Glaucoma, non-glaucoma	Fundus	110 images		6 ophthalmologists	Pegasus	AUC: Pegasus: 92.6%, ophthalmologist: 69.6% -84.9%, the "best case" consensus scenario AUC of 89.1%. Sensitivity: Pegasus: 83.7%; ophthalmologists: 61.3%-81.6%. Specificity: Pegasus: 88.2%, ophthalmologists:80.0% -94.1% Agreement with gold standard: Pegasus: 0.715; highest ophthalmologist: 0.613.	Pros: Pegasus outperformed 5 of the 6 ophthalmologists in terms of diagnostic performance, and there was no statistically significant difference between the deep learning system and the “best case” consensus between the ophthalmologists. Cons: small sample size and fundus from same camera.
Norouzifard et al. [102]	Glaucoma diagnosis	Glaucoma, normal	Fundus	447 images	HRF (30 images)		Inception-ResNet-V2	accuracy: 80%	Pros: validated on independent dataset. Cons: both training and validation datasets were small.
Shibata et al. [105]	Glaucoma screening	Glaucoma, normal	Fundus	3132 images (1364 glaucoma, 1768 normal)	110 images (60 glaucoma, 50 normal)	3 residents	ResNet	AUC: model:96.5% (93.5–99.6%); residents: 72.6–91.2%.	Pros: the performance was compared with human expert and the model outperformed them. Cons: the current study excluded photographs with features that could interfere with an expert diagnosis of glaucoma, which was not “real world” setting.
Li et al. [256]	VF classification	Glaucoma, non-glaucoma	VF (VF PD images)	4012 VFs	300 VFs	9 ophthalmologists	CNN	Model: accuracy: 0.876 (95% CI 0.838–0.914); sensitivity: 0.932; specificity: 0.826 Ophthalmologists: the average accuracies are 0.607, 0.585 and 0.626 for resident ophthalmologists, attending ophthalmologists and glaucoma experts, respectively.	Pros: CNN has achieved higher accuracy compared to human ophthalmologists and traditional rules; Cons: used only pattern deviation images as the input of CNN, preperimetric glaucoma may not be effectively detected by machine.
Andersson et al. [75]	Glaucoma Diagnosis	Glaucoma, healthy	VF (30–2 VF)	165 subjects (99 glaucoma,66 healthy)		30 physicians	ANN	ANN: sensitivity: 93%, specificity: 91% Physicians: sensitivity: 61%- 96% (mean 83%); specificity:59%-100% (mean 90%).	Pros: ANN performs at least as well as physicians in assessments of visual fields for the diagnosis of glaucoma; Cons: external and larger datasets are needed for further validation.
Goldbaum et al. [12]	Glaucoma diagnosis	Glaucoma, normal	VF (24–2 VF) + age	345 eyes (189 normal, 156 glaucoma)		2 human experts	Mixture of Gaussian (MoG)	MoG had significantly greater ROC area than PSD and CPSD. Human experts were not better at classifying visual fields than the machine classifiers or the global indices. MoG(PCA): AUC: 0.922; sensitivity: 0.67(at specificity = 1), 0.79 (at specificity = 0.9); Expert 1: sensitivity = 0.75, specificity = 0.96; Expert 2: sensitivity = 0.88, specificity = 0.59.	Pros: compared multiple methods based on different parameters. Cons: larger and external validation are needed for further evaluation.
Goldbaum et al. [63]	Glaucoma diagnosis	Glaucoma, normal	VF	120 eyes (60 normal, 60 glaucoma)		2 glaucoma specialists	ANN (2 layered)	The experts and the network were in agreement about 74% of the time ANN: accuracy: 67%; sensitivity: 65%; specificity: 71%; glaucoma specialists: accuracy: 67%; sensitivity: 59%; specificity: 74%.	Pros: compared with human expert. Cons: dataset was small, performance needs to be improved and external validation was needed.

It is also challenging to compare the performance of different AI models as they typically used differing definitions of glaucoma, different methodologies to develop models and various strategies for labeling, as well as images from glaucoma patients at varying levels of severity. In addition, they typically employed images with diverse quality under different instrument settings and included different populations from primary, secondary, or tertiary centers. As a result, for example, it is unfair to compare a model trained on normal subjects and glaucoma patients at the moderate to advanced stages of the disease with another model trained on a dataset with patients at a wider spectrum of glaucoma severity. Model reliability is also a barrier for real-life clinical applications, as most DL models are deterministic and provide an output regardless of whether the input image is even relevant. Finally, most DL models provide a “black box” architecture without appropriate visualization and interpretability, thus lowering user trust and creating another challenge for a successful integration.

## Future directions

Two autonomous AI models have been approved by the FDA to detect more than mild diabetic retinopathy and diabetic macular edema [302]; however, there is no FDA-approved autonomous AI instrument for detecting glaucoma. Therefore, the development of innovative AI models to autonomously assess and detect glaucoma remains an important goal for improving treatment outcomes for this major blinding disorder. To achieve this goal, we suggest implementing the following directions in the future studies:

Lack of consistent and objective definitions of glaucoma and its progression leads to improper AI model evaluations. As such, addressing this challenge is a primary step to improve integration of AI glaucoma research and clinical applications. One potential solution may be using quantified parameters from VF and OCT data to create objective criteria for glaucoma definition. More recently, some groups have worked on identifying more objective criteria for defining GON and glaucoma staging based on OCT and VFs [87, 303, 304]. However, this active area of research requires further studies. Firstly, these criteria need to be validated further based on larger and more diverse datasets. Secondly, data collected from different OCT or VF instruments may impact the identified objective criteria. Lastly, comorbidity and ethnic-specific criteria may influence findings thus considering the effect of these parameters is vital. The other area that needs attention is the architecture of the AI models. In particular, DL models sometimes can be fragile, unexplainable, and non-interpretable. Many teams are currently working on these limiting aspects of the DL models and hopefully some limitations will be addressed in the near future. New studies may incorporate interpretability into the AI model through various techniques, such as feature importance, SHAP value, Grad-CAM, saliency map, and Poly-CAM. Some current studies have already included interpretability elements like feature importance and Grad-CAM. Thakoor et al. [279] developed end-to-end CNN architectures to detect glaucoma based on OCT images. They applied Grad-CAM and tested with concept activation vectors (TCAVs) to infer what image concepts CNN models rely on to make predictions and compared with that of human experts by tracking eye fixations. They identified consistent regions of OCT are evaluated based on CNN and OCT experts in detecting glaucoma. Such studies can shed lights on improving interpretability of AI models by applying multiple consistent methods and comparing with clinicians’ eye fixations. Future work may validate these studies using more diverse datasets. Also, comparative studies can be conducted based on multiple data modalities by including or masking human focused region to indirectly identify areas that are more important for CNN models.

Another practical approach to partially address some of these limitations is to perform accurate quantification of CDR in fundus and RNFL thickness profiles in OCT images to enhance performance and improve interpretability. Moreover, longitudinal assessment of these clinically relevant quantified parameters may allow a more consistent and accurate monitoring and progression detection.

Another future direction could be improving datasets for training AI models. Some of the reference datasets are annotated by non-glaucoma experts thus may not represent GON accurately. Therefore, generating more accurate reference datasets with panels of glaucoma specialists are warranted. We also suggest selecting diverse populations of patients (for instance, datasets for population-based screening and diagnosis for intended use) from different ethnicities across all glaucoma severity levels to minimize selective bias. Due to the complex nature of glaucoma (e.g., different disease processes can lead to the same optic nerve degeneration, glaucoma can look and progress differently due to different disease mechanisms, glaucoma has several phenotypes such as open angle, closed angle, secondary glaucoma, low tension, etc.), making it difficult to standardize outcomes of glaucoma studies across different investigations, we strongly recommend generating multi-modal glaucoma datasets given the fact that glaucoma is multi-factorial and complex, thus several modalities may enhance detection and monitoring tasks. However, obtaining a sufficiently large dataset with several modalities can be challenging. A primary priority for future AI studies could be implementing guidelines regarding designing and reporting AI studies [305, 305–307] to minimize evaluation and comparison challenges.

Another promising solution is developing foundational model using self-supervised learning [308] based on known diverse datasets to improve the performance and generalizability. This is particularly important given the fact that the performance of the AI models can vary depending on the severity level of the patients with glaucoma in the dataset, as AI models may perform better on datasets with a greater number of patients at the advanced level of glaucoma compared to datasets with a greater number of patients at the early stages of glaucoma.

Improving dependability of AI models can build trust. One direction could be developing probabilistic DL models [231] that can generate the likelihood as well as the level of confidence of the model on generated outcome. This way, clinicians have two levels of outcome to make a final decision and thus may trust the class of AI models better. However, probabilistic models are more challenging to develop due primarily to more complexity that typically leads to suboptimal performance, thus only a few studies have utilized probabilistic models. Recent CNN architectures such as ConvNeXt [309] and availability of larger datasets may however address these challenges.

The paradigm of applications of AI in glaucoma changed in 2016 with applications of deep CNN models in glaucoma. [310]. However, we believe a second major impact will result from the application of ChatGPT [311], first initiated in late 2022. ChatGPT can be a great tool for many different aspects of glaucoma, and a recent study showed that ChatGPT can assist glaucoma diagnosis based on clinical case reports and obtained comparable performance with senior ophthalmology residents [312]. We believe the development of large language models (LLMs) with glaucoma domain-specific knowledge that leverage multi-modal data in combination with active learning holds more promise for future integration into clinical practice.

## Conclusions

The advancement of AI in glaucoma detection and monitoring is progressing rapidly. In recent years, numerous innovative DL models have been developed specifically for diagnosing glaucoma, showcasing remarkable performance. However, despite their promising results, none of these models have received FDA approval for being used in glaucoma clinical practice. This is partly due to obstacles such as inconsistencies in defining glaucoma, the generalizability and reliability of the models, and their interpretability. To enhance the integration of these technologies into healthcare settings, future research is essential to address these potential challenges, including generation of dependable gold standards, improving model generalizability, reliability, interpretability as well as legal, ethical, and patient privacy issues, among several others. Successful integration of AI in glaucoma clinical practice, and ophthalmology, requires addressing challenges facing all elements of the healthcare pipeline.

## Methods

**Fig. 4:**
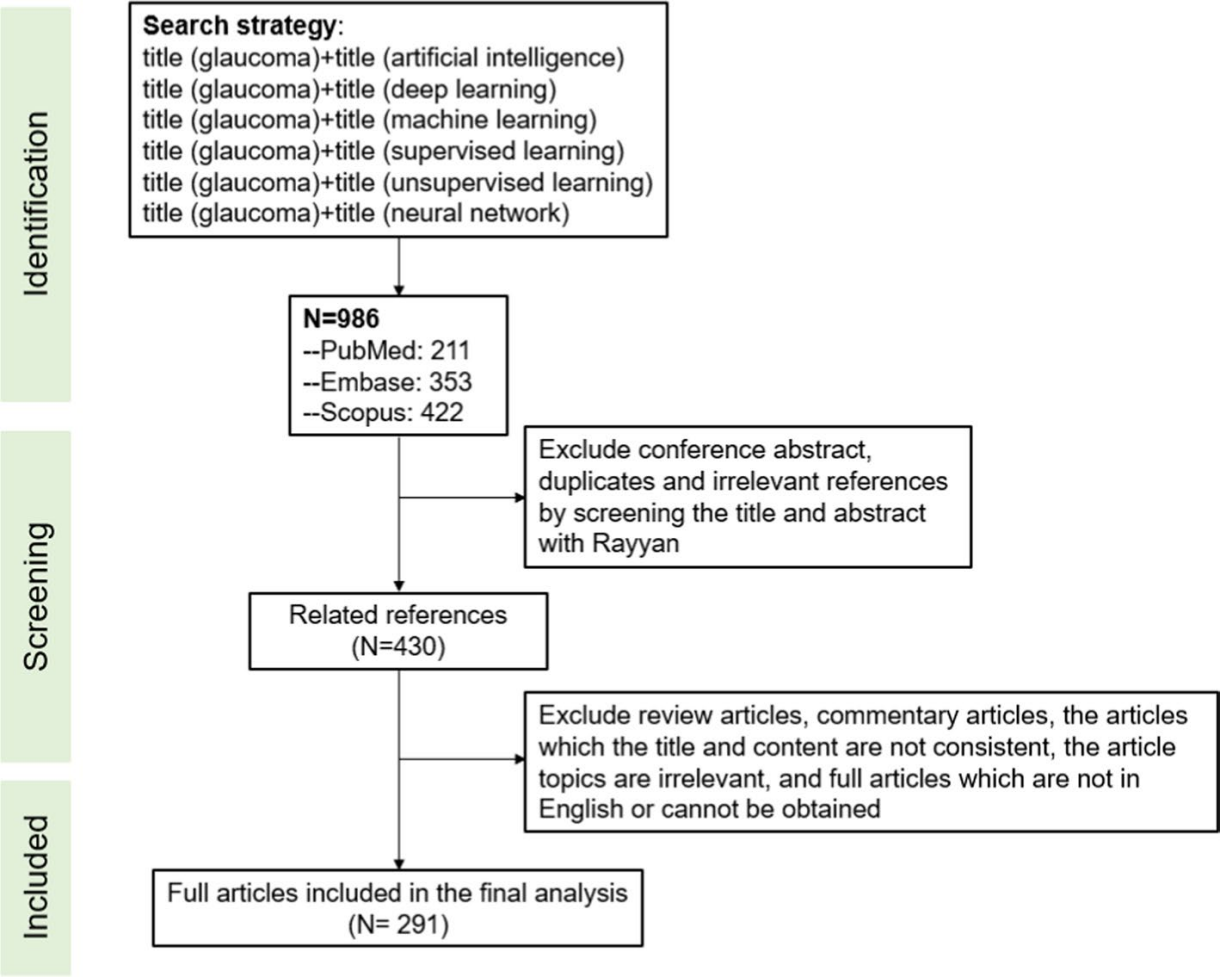
Proposed review methodology for sample collection and analysis

We searched PubMed, Scopus, and Embase databases for the AI-related studies in glaucoma conducted through 2022. Figure 4 shows the methodology utilized in this review. We first used “glaucoma”, “machine learning”, “deep learning”, “artificial intelligence”, and “neural network” keywords and searched through the title of papers in these three databases to identify related papers. Our initial search identified 986 articles. We then screened the collected articles for duplicates and irrelevant topics and included 430 relevant and unique papers. Finally, we went through the remaining articles and excluded 139 articles that were review papers, or the contents were irrelevant, or essential information (number of samples) was missed, the full texts were not in English or the full text cannot be obtained. The final study included 291 full-text articles discussing various applications of AI in glaucoma.

## Data Availability

No new data were created or analyzed in this study. Data sharing is not applicable to this article.
